# The correlation of whole blood viscosity and outcome in mechanical thrombectomy for acute ischemic stroke

**DOI:** 10.3389/fstro.2025.1517343

**Published:** 2025-03-12

**Authors:** Monika Thapa, Jordyn Courville, Reese Leonhard, Prabandh Buchhanolla, Mohammad Abdurrehman Sheikh, Rahul Shah, Prashant Rai, Himanshu Chokhawala, Md Ismail Hossain, Mohammad Alfrad Nobel Bhuiyan, J. Dedrick Jordan, Roger E. Kelley

**Affiliations:** ^1^Department of Neurology, Louisiana State University Health Shreveport, Shreveport, LA, United States; ^2^Biostatistics and Computational Biology Laboratory, Department of Medicine, Louisiana State University Health Sciences Center, Shreveport, LA, United States

**Keywords:** stroke, ischemia, thrombectomy, viscosity, large vessel occlusion, modified Rankin score

## Abstract

**Introduction:**

Whole blood viscosity (WBV), reflecting the intrinsic resistance of blood flow, is an established predictor of stroke events in individuals. This study aims to correlate the WBV at different shear rates with the outcome of mechanical thrombectomy, known to be an effective treatment for large vessel occlusion (LVO) stroke.

**Method:**

This is a single-center retrospective study conducted at our comprehensive stroke center. The charts of 317 patients who underwent mechanical thrombectomy within 6 h of LVO stroke presentation were reviewed. The modified Rankin score (mRS) at discharge was used as the outcome measure, with individuals categorized as low (0–2) or high (3–6). WBV at different shear rates was calculated using De Simone's Formula. The *T*-test and Chi-square test were used to compare baseline continuous and categorical data, respectively, amongst the mRS study groups. We utilized multivariable logistic regression analyses to identify the independent risk factors associated with the outcome of interest following mechanical thrombectomy. In addition, Spearman rank order correlation was used to assess for r value between mRS and WBV at different shear rates.

**Results:**

Baseline group characteristics, including demographics and medical history, were similar among the two study groups. Of note, our study found no significant differences in clinical outcomes between the two groups with WBV at high shear rate (OR 0.969, 95% CI 0.77–1.204, *p* = 0.780) and low shear rate (OR 0.998, 95% CI 0.988–1.008, *p* = 0.779) following mechanical thrombectomy. Spearman rank order correlation between mRS at discharge with WBV at high shear rate (*r* = 0.058, *p* = 0.123) and low shear rate (*r* = 0.048, *p* = 0.128) was non-significant.

**Discussion:**

There is limited information of the effect of WBV at high and low shear rates on the clinical outcome following mechanical thrombectomy in patients with LVO. Our results revealed that WBV at high and low shear rates did not impact the functional outcome of mechanical thrombectomy. This result might be affected by the potential limitation of the formula used to derive the given shear rates. Despite this lack of association observed in our study, other contributors of viscosity may still potentially play a significant role in outcome following mechanical thrombectomy.

## Introduction

Whole blood viscosity (WBV) is a measure of resistance of blood to flow, resulting from friction between adjacent layers of blood (Pop et al., 2002). As a non-Newtonian fluid, the viscosity of blood is dependent on a variable range of shear rates or shear stress. Moreover, blood is a tissue composed of different cell types (e.g., RBCs, WBCs platelets, and plasma; Baskurt and Meiselman, 2003). Thus, the determinants of blood viscosity can be postulated as hematocrit, plasma viscosity (itself determined by lipoproteins and plasma fibrinogen), red cell aggregations, and red cell deformation (Lowe et al., 1997). In 1989, Simone et al. identified the hematocrit as the most important contributor to blood viscosity and derived an equation to calculate WBV using hematocrit and total plasma protein level, the latter an indicator of plasma viscosity (De Simone et al., 1990).

Disorders in the hemorheological variables, including blood viscosity, have been shown to constitute a significant risk factor in both the development and exacerbation of cardiovascular disorders (Tekin Tak et al., 2020; Cecchi et al., 2009). Prior studies have suggested the role of elevated blood viscosity to be a stronger predictor of stroke, and its recurrence, than more conventional risk factors (Lowe et al., 1997; Velcheva et al., 2008).

Mechanical thrombectomy (MT) has revolutionized the approach to acute stroke due to LVO (Berkhemer et al., 2015). Following the introduction of MT, further studies have continued to demonstrate its therapeutic efficacy for patients with LVO (Ghozy et al., 2022), in the context of improving degree of disability and early neurologic recovery with better functional outcomes (Saver et al., 2016). However, excellent outcomes are not routine, and prognosis presumably reflects coexistent factors such as age and timing of the procedure; additionally, intrinsic factors such as hemorheological status also play a role in the clinical outcome of patients with acute ischemic stroke (Costalat et al., 2012; O'Connor et al., 2020). The modified Rankin scale (mRS) is a well established outcome measure for stroke patients, which grades a patient's disability from 0 (no symptoms) to 6 (death; Powers et al., 2019; Banks and Marotta, 2007).

In our study, we assessed for a potential correlation between WBV at two different shear rates with the outcome following MT based on mRS scores at discharge in patients with LVO. It was proposed that our findings might provide relevant information about the effect of WBV on successful endovascular intervention in strokes secondary to LVO.

## Methods

This was a single center retrospective study conducted at our comprehensive stroke center from January 2018 to July 2023, with inclusion of 317 consecutive patients in total. These were patients aged 18 years to 89 years who had undergone MT for LVO, with or without intravenous thrombolytics, within 6 h of stroke onset. Patients with any cancer, severe anemia, and those with incomplete information from the database were excluded. The study was approved by the local Institutional Review Board (IRB) of the medical center.

Per conventional outcome measures employed in analyses of patients with strokes, the study population was divided into two groups based on mRS score at discharge following MT. Group 1 is comprised of patients with mRS score 0–2 (favorable outcome) and Group 2 with mRS score 3–6 (unfavorable outcome). The WBV for each patient at high shear rates (HSR) and low shear rates (LSR) was calculated using De Simone's Formula: (De Simone et al., 1990).


$$\begin{array}{l} WBV\;at\;HSR\;(208/sec-1)=(0.12\;x\;HcT)+0.17\;(TP-2.07)\\ WBV\;at\;LSR\;(0.5/sec-1)=(1.89\;x\;HcT)+3.76\;(TP+78.42)\\ \;where\;TP\;is\;total\;protein\;in\;g/L,HcT\;is\;hematocrit\;in\;\% \end{array}$$

Data analysis was performed using R software. For the comparison of continuous data across two groups, the *t*-test was chosen as the most appropriate statistical test. Similarly, given that categorical data were being compared across *n* = 2 groups, the Chi-square test was deemed appropriate for this purpose.

Given a binary outcome variable, that of two mRS score groups (i.e., 0–2 vs. 3–6), logistic regression was selected as the most appropriate statistical methodology for model analysis. Importantly, this was performed in both a univariate and more rigorous multivariable fashion. Per convention, a *p*-value < 0.05 was used as statistically significant. Finally, given the underlying non-parametric distribution of the data, Spearman rank order correlation was used to assess for *r* between mRS and WBV at different shear rates, as opposed to a more traditional Pearson's *r*.

## Results

We analyzed the data of 317 patients from the database. The mean age + SD in group 1 was 66.8 ± 13.7; in group 2, this was 66.7 ± 14.4. In group 1, the female population was 41%, whereas in group 2, this was 47%. There was no significant difference in age (*p* = 0.97) and gender (*p* = 0.38) between the two groups. Fifty-seven percentage were found to have a smoking history in group 1, greater than those found in group 2, 44%; this difference was statistically significant (*p* = 0.046). Time from door to puncture site was less in group 1 compared to group 2 (101 min vs. 121 min, *p* = 0.01). There were no significant differences between the two groups in terms of NIHSS at presentation, pre-existing diabetes mellitus, hypertension, coronary artery disease, atrial fibrillation, or laboratory findings of hematocrit, platelet, total protein, and lipid profile (Table 1).

**Table 1:** Demographic and medical history summary.

**Variables**	**mRS 0–2 (88)**	**mRS 3-6 (229)**	**p-value**
Age (years)	66.8 ± 13.7	66.7 ± 14.4	0.97
Gender (%)			0.38
Female	41	47	
Male	59	53	
NIHSS at presentation	13.2 ± 6.76	17.1 ± 6.69	0
Prior stroke (%)	16	17	1
BMI	31.2 ± 7.69	30.2 ± 8.09	0.33
Hypertension (%)	79	86	0.17
DM (%)	21	32	0.62
Coronary artery disease	15	20	0.40
Atrial fibrillation	30	27	0.75
Smoking	57	44	0.04
Hematocrit	0.39 ± 0.06	0.39 ± 0.07	0.79
Platelet	236 ± 82.6	247 ± 84.3	0.29
Total protein	67.9 ± 7.03	68 ± 7.45	0.9
Total cholesterol	154 ± 38.5	160 ± 44.8	0.24
TG	108 ± 60.8	118 ± 84.2	0.3
HDL	44.6 ± 13.3	45.9 ± 16.6	0.46
LDL	89.2 ± 32	93.4 ± 39.0	0.36
Door to puncture site	101 ± 51.6	121 ± 84.6	0.01
Reperfusion injury after MT(%)	12	21	0.11
WBV at HSR	11.2+-1.2	11.2+-1.25	0.97
WBV at LSR	−38.8+-26.5	−38.6+-27.7	0.97

NIHSS, National Institutes of Health Stroke scale; BMI, Body mass index; DM, Diabetes mellitus; TG, Triglycerides; HDL, high density lipid; LDL, Low density lipid; mRS, modified Rankin scale; MT, mechanical thrombectomy; WBV, whole blood viscosity; HSR, high shear rate; LSR, low shear rate.

Our study found no significant differences for a positive clinical outcome (mRS at discharge of 0–2) with WBV at HSR (OR 0.969, 95% CI 0.77–1.204, *p* = 0.780) and LSR (OR 0.998, 95% CI 0.988–1.008, *p* = 0.779) following mechanical thrombectomy.

**Figure 1:**
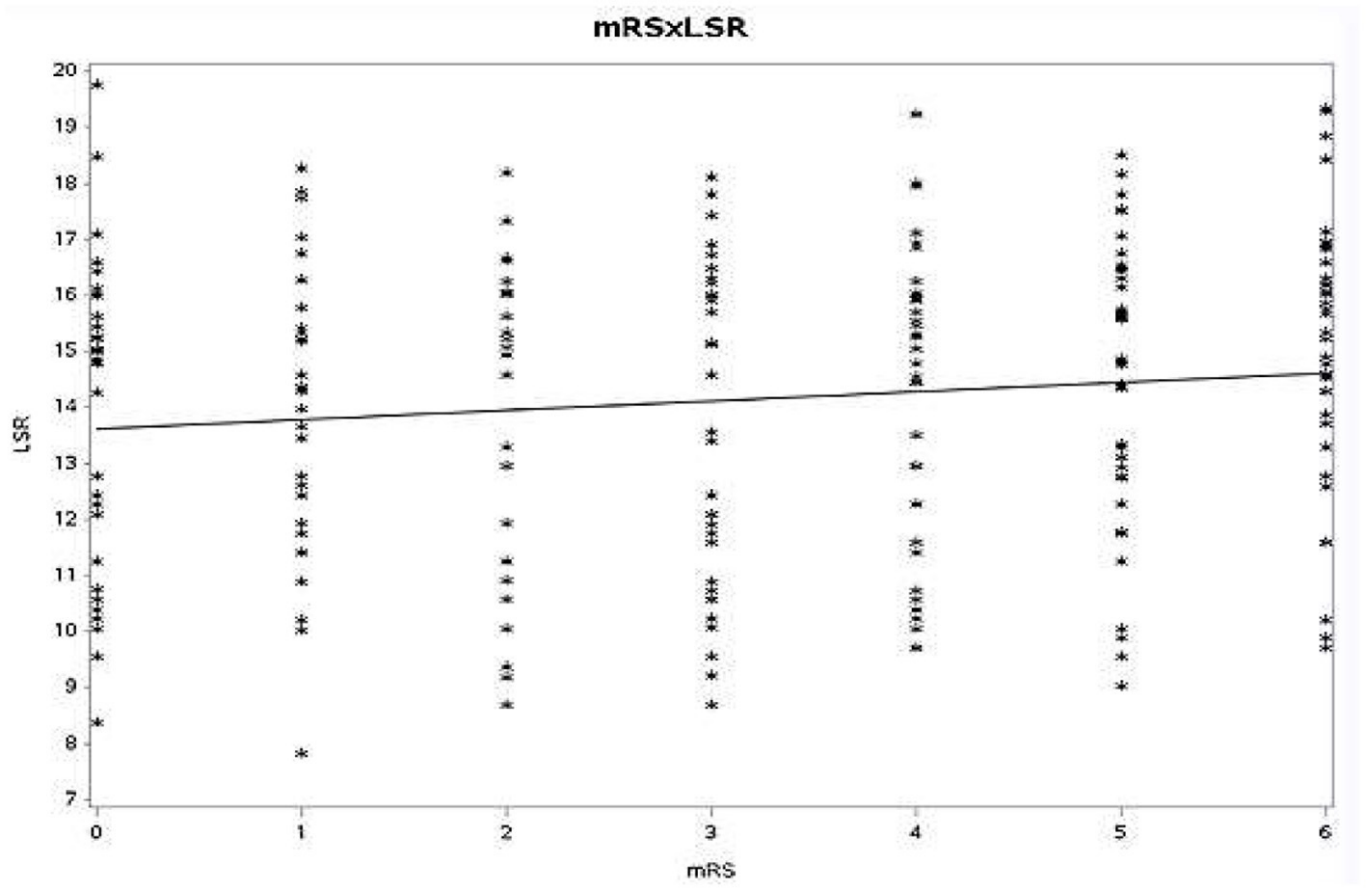
Spearman rank-order correlation between mRS and LSR (mRS, modified Rankin scale; LSR, low shear rate).

**Figure 2:**
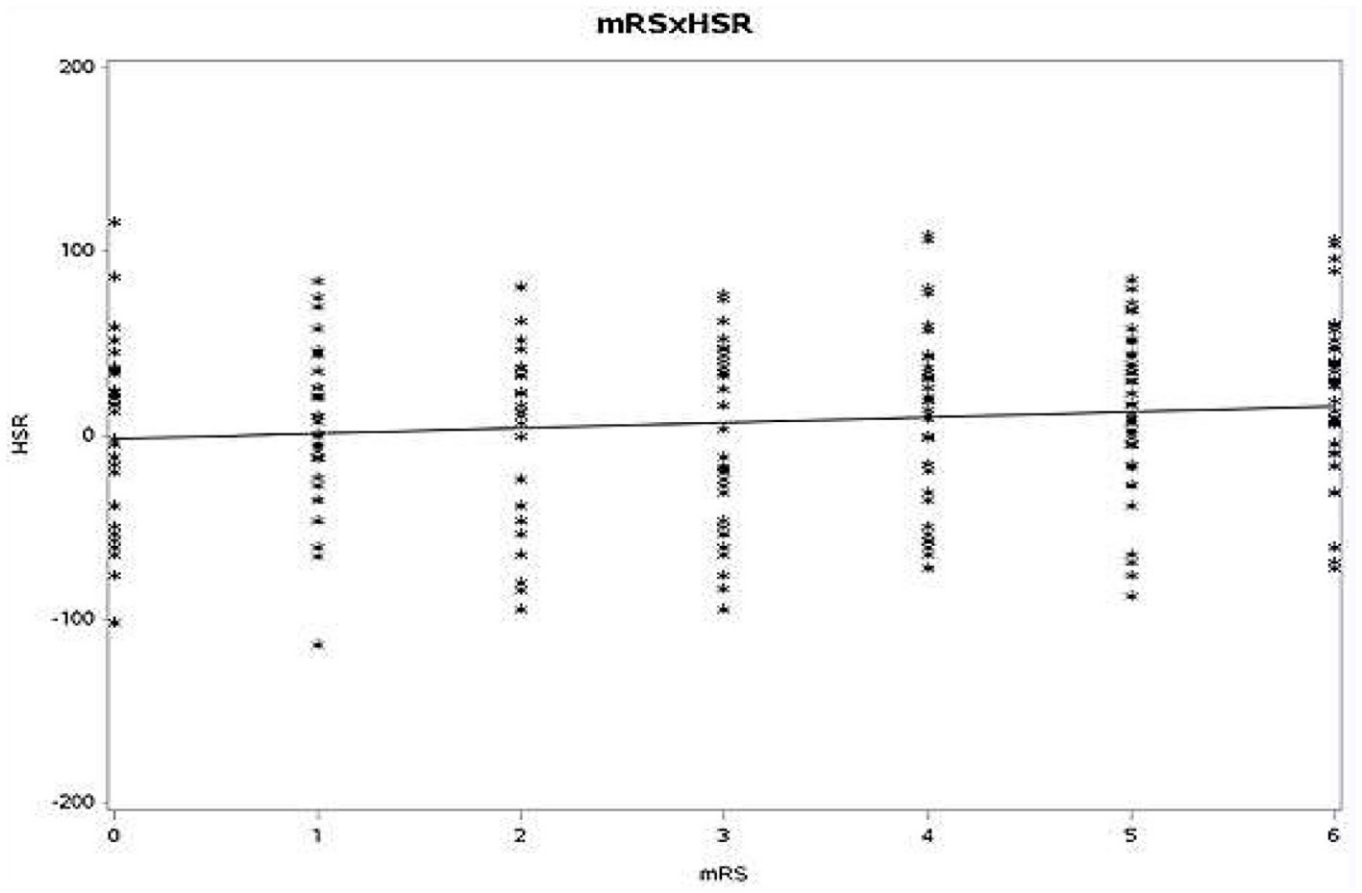
Spearman rank-order correlation between mRS and HSR (mRS, modified Rankin scale; HSR, high shear rate).

Spearman rank order correlation between mRS at discharge with WBV at HSR (*r* = 0.058, *p* = 0.123) and LSR (*r* = 0.048, *p* = 0.128) was also non-significant (Figures 1, 2).

## Discussion

Based on the analysis conducted using the present dataset, we did not find any correlation between WBV at high vs. low shear rates and clinical outcomes following MT. This result is in concordance with a study conducted by Hashem et al., where significant correlation between an indicator of blood viscosity and stroke outcomes based on NIHSS and mRS scores was not observed. However, they reported relationship between both hematocrit and albumin, and stroke outcome in their study (Hashem et al., 2018).

Several other hematological factors have been shown to be associated with clinical and functional outcomes following acute ischemic stroke. Endothelial shear stress, proportional to blood viscosity, is a potent local stimulus for the formation and progression of atherosclerotic plaque (Chatzizisis et al., 2007). Studies conducted by Lowe et al. (1997) revealed a well-established association between endothelial sheer stress and a majority of cardiovascular events, including stroke and ischemic heart disease. WBV at both HSR and LSR is also associated with acute arterial occlusion, triggering a sudden decrease of blood flow to the area supplied by the affected artery (Erdogan et al., 2020; Çekici et al., 2019; Li et al., 2015). Increased fibrinogen, a determinant of plasma viscosity, and blood viscosity have also been shown to significantly contribute to the clinical outcome in patients who suffered from stroke (Resch et al., 1992).

The effects of MT on clinical outcomes is well-established in the literature. However, certain studies have also shown that successful reperfusion following MT appears to not necessarily correlate with positive clinical outcomes. The gap is well demonstrated in the literature in the difference in percentage of patients achieving successful reperfusion and the percentage of those achieving mRS 0–2 scores by 15–28%(Grotta and Hacke, 2015). Other different predictors of poor outcome that have been demonstrated, in the literature include age, site of occlusion, NIHSS score, history of diabetes mellitus, TICI score, number of passes, use of tPA, hematocrit, and serum albumin (Hashem et al., 2018; Linfante et al., 2016; Gordon et al., 2018).

Hemorheological abnormalities such as increases in WBV and plasma viscosity were demonstrated in the development of acute cerebral ischemia in study conducted by Fisher and Meiselman (1991). High blood viscosity is associated with increased thromboembolic risk and correlated with systemic inflammation as well (Pop et al., 2002).

Given the above, one would expect a relationship between WBV and clinical and functional stroke outcome. However, WBV, which directly and indirectly affects stroke incidence and outcomes, has rarely been identified as a predictor of thrombectomy outcome. In a study, Yenerçag et al. looked at the association of WBV with clinical outcome following MT in patients with acute ischemic stroke (Yenerçag et al., 2021), the authors reported that an increased WBV is an independent risk factor and is correlated with poor clinical outcomes in acute ischemic stroke patients treated with MT (Yenerçag et al., 2021). This is in contrast with the findings of our study. Despite a relatively large patient population, the retrospective nature of the study introduces the possibility of selection bias, where the included patients may not accurately represent the broader population. Variations in blood collection times across patients can significantly impact laboratory values. They might also be affected due to preexisting comorbidities with medication history in most of the patients as the retrospective design hindered the accurate determination of diagnosis timing for these factors. Most studies have used viscometer which provide an accurate estimate of blood viscosity compared to the formula we used in our study (Kensey, 2003; Cowan et al., 2012). Additionally, although objective measures were assed, any errors in mRS adjudication may introduce errors of outcome misclassification into the analysis as well. Finally, in using traditional logistic regression, although per prevailing convention in stroke analyses, more granular information is not discerned compared to, for example, the use of an ordered logit model.

Taking into account our findings, the relationship between functional outcome and WBV following MT requires further study. We believe that our attempt to study the correlation between a complex biomarker and stroke and the limitations provides valuable insights and lays the groundwork for future research with improved methodologies and a more robust design.

## Conclusion

Though a prior study has demonstrated the role of WBV on stroke outcomes, the evidence for an effect on clinical and functional outcomes following MT has been less robust. The present study did not find an association between WBV at HSR and LSR and mRS at discharge, in agreement with most previous work exploring this association. However, the presence of underlying associations between WBV and stroke outcomes, as well as work by Yenerçag et al., suggests that further study is needed to more thoroughly explore these potential associations.

## Data Availability

The raw data supporting the conclusions of this article will be made available by the authors, without undue reservation.
